# Kuhuang alleviates liver fibrosis by modulating gut microbiota-mediated hepatic IFN signaling and bile acid synthesis

**DOI:** 10.3389/fphar.2022.1080226

**Published:** 2022-12-13

**Authors:** Bo Shen, Cui Zhou, Tianyi Gu, Zhenyang Shen, Yuecheng Guo, Weiming Dai, Yang Liu, Jie Zhang, Lungen Lu, Hui Dong

**Affiliations:** ^1^ Department of Gastroenterology, Shanghai General Hospital, Shanghai Jiao Tong University School of Medicine, Shanghai, China; ^2^ Suzhou Leiyunshang Pharmacology Group, Shanghai, China

**Keywords:** Kuhuang, liver fibrosis, bile acids, interferon, gut microbiota

## Abstract

**Background:** Liver fibrosis is a common outcome of the pathological progression of chronic liver disease; however, no specific and effective therapeutic agent has been approved for its treatment. We investigated the effects of Kuhuang on liver fibrosis and the underlying mechanisms of action.

**Materials and methods:** To induce hepatic fibrosis, either 3,5-diethoxycarbonyl-1,4-dihydro-collidine (DDC) diet was administered, or bile duct ligation (BDL) surgery was performed on C57BL/6 mice. Kuhuang was orally administered to mice for 7 days before and after bile duct ligation or 4 weeks with a DDC diet. Hematoxylin and eosin, Sirius red staining, and immunohistochemical analyses were performed to evaluate hepatic pathology. Hepatic interferon-β (IFN-β) levels were measured using an enzyme-linked immunosorbent assay. RNA sequencing was performed to examine the gene expression profiles of liver tissues. The mRNA expression of inflammatory, profibrotic, and bile acid (BA)-related genes was further validated by qRT-PCR. A targeted metabolomics assay revealed the alteration of the hepatic bile acid (BA) composition. The composition of the gut microbiota was determined *via* 16S rRNA sequencing.

**Results:** Treatment with Kuhuang attenuated liver fibrosis and reduced the inflammatory response in bile duct ligation and DDC mouse models. In addition, the hepatic IFN signaling pathway was activated following Kuhuang treatment. Kuhuang treatment also significantly decreased hepatic levels of both primary and secondary BAs. In addition, Kuhuang treatment altered gut microbiota composition, with an increased abundance of interferon-inducing *Akkermansia* and decreased abundance of bile salt hydrolase-producing *Lactobacillus*, *Clostridium,* and *Bifidobacterium*. Furthermore, the abundance of *Akkermansia* was positively correlated with the hepatic mRNA expression levels of *Ifna4, Ifnb,* and *Isg15,* whereas that of *Lactobacillus*, *Clostridium*_*-*_ *sensu*_*-*_ *stricto*_*-*_ *1,* and *Bifidobacterium* was positively correlated with levels of bile acid synthesis-related genes.

**Conclusion:** Our results suggest that Kuhuang plays a protective role during the progression of liver fibrosis, potentially by altering the composition of the gut microbiota, which consequently activates interferon signaling and inhibits bile acid synthesis in the liver.

## Introduction

Liver fibrosis, characterized by excessive extracellular matrix deposition, is a common pathological process in various chronic liver diseases, including viral hepatitis, alcohol-related liver disease, non-alcoholic fatty liver disease, autoimmune hepatitis, cholestatic liver disease, drug-related hepatitis, and genetic diseases (Guo et al., 2022b). Persisting and iterating liver fibrosis tends to develop into cirrhosis, a severe health burden globally (Ginès et al., 2021). However, no specific and effective therapeutic agents have been approved to treat liver cirrhosis. Fortunately, in rare cases, fibrosis may be reversible (Lee et al., 2015). Therefore, discovering drugs for liver fibrosis therapy is urgent to meet this clinical need, considering the high morbidity and mortality associated with chronic liver disease.

Kuhuang, a traditional Chinese herbal compound—composed of Yin Chin, Da Huang, Ku Shen, Da Qingye, and Chai Hu—has been proven clinically effective in treating cholestasis for many years (Xu et al., 2009). Previous studies have provided convincing evidence for the protective role of Kushen, an important component of Kuhuang, in liver fibrosis and carcinoma. In a model of liver fibrosis, the compound Kushen injection (CKI) repressed hepatic stellate cell (HSC) activation and protected against hepatocellular carcinoma by regulating TGFβ/Smad7 signaling (Yang et al., 2021). In addition, Yang et al. (2020) revealed that CKI could alleviate immunosuppression in the tumor-associated microenvironment by activating proinflammatory responses, suggesting that Kushen might also participate in regulating inflammatory responses. However, as Kuhuang is composed of multiple components besides Kushen, other mechanisms must be involved in its ability to alleviate liver fibrosis. To date, the mechanisms of action of Kuhuang in liver disease have not been elucidated.

Bile acids (BAs), the main constituents of bile, not only function as important digestive fluids but also as essential signaling molecules. When exposed to excessive BAs, bile ducts and hepatocytes are prone to injury, accompanied by mitochondrial dysfunction and recruitment and infiltration of inflammatory cells (Li et al., 2017; Trauner et al., 2017; Zhang et al., 2019a). BAs play an essential role in HSC proliferation and activation (Svegliati-Baroni et al., 2005; Liu et al., 2018). Constant ductular injury, enduring inflammation, and HSC activation eventually lead to liver fibrosis (Liu et al., 2018). In the liver, the classic pathway of BA synthesis is regulated by the BA-activated farnesol X receptor (Fxr), which subsequently facilitates slight heterodimer partner-dependent inhibition of cholesterol 7α-hydroxylase (encoded by *CYP7A1*) (Lu et al., 2000). In addition, activated Fxr-FGF15/19 signaling in the ileum suppresses BA synthesis through *CYP7A1* (Katafuchi and Makishima, 2022). The gut microbiota is another crucial modulator in controlling BA homeostasis by linking the circulation of BAs between the liver and gut (Jiang et al., 2022). Bile salt hydrolase (BSH) activity is essential for the deconjugation of primary BAs by gut microbiota (Mori et al., 2022) and is mostly facilitated by Gram-positive bacteria, such as *Lactobacillus* (Jayashree et al., 2014), *Bifidobacterium* (Kim et al., 2005), *Enterococcus* (Wijaya et al., 2004), and *Clostridium* spp. (Rossocha et al., 2005) as well as in commensal Gram-negative *Bacteroides* spp. (Long et al., 2017). In turn, BAs are crucial players in modulating the composition of gut microbiota (Xu et al., 2022). Targeting the gut microbiota–BA axis may be a promising therapeutic strategy for liver fibrosis.

Although Kuhuang has been proven to be effective in alleviating cholestasis, the underlying mechanism remains unclear. To explore the mechanism of Kuhuang, we performed hepatic transcriptomics and targeted metabolomics analyses, as well as 16S rRNA sequencing of fecal samples in a mouse model of cholestasis. We found that Kuhuang could alleviate hepatic inflammatory response, BA accumulation, and liver fibrosis. The potential mechanism of Kuhuang may involve the activation of interferon signaling and inhibition of BA synthesis by altering gut microbiota composition.

## Materials and methods

### Animal models of DDC and BDL

A total of 25 male C57BL/6J mice (8 weeks old) were divided randomly into five groups (n = 5 per group): Chow group fed a chow diet; 3,5-diethoxycarbonyl-1,4-dihydro-collidine (DDC)+Kuhuang (KH) group fed a DDC diet (Trophic Animal Feed High-tech Company, Nantong, China) plus oral KH administration; DDC + H_2_O group fed a DDC diet plus oral H_2_O administration; bile duct ligation (BDL)+KH group treated with BDL under anesthetic condition plus oral KH administration, and BDL + H_2_O group treated with BDL plus oral H_2_O administration. For the BDL model (*n* = 5 per group), Kuhuang dissolved in ddH_2_O (20 mg/g body weight, BDL + KH group) (Zhang et al., 2019) or ddH_2_O (BDL + H_2_O group) was administered orally every day for 7 days before and after BDL, respectively. For the DDC model (*n* = 5 per group), Kuhuang (DDC + KH group) or ddH_2_O (DDC + H_2_O group) were administered orally every day for 4 weeks along with a DDC diet. The chow group (*n* = 5) received no intervention. Kuhuang granules were provided by Suzhou Leiyunshang Pharmacology Group (Suzhou, China). Animals were maintained in a specific pathogen-free environment with unrestrained access to food and water. The Animal Ethics Committee of the Shanghai General Hospital, Shanghai Jiaotong University School of Medicine, approved all animal-related experiments. Mice were treated with 3% phenobarbital sodium before sacrifice. Blood, liver, and fecal samples were collected. Liver tissues were either stored at −80°C for protein and RNA extraction or fixed with formalin, followed by paraffin embedding for histopathology analysis.

### Quantitative real-time reverse-transcriptase polymerase chain reaction

Total RNA was isolated from cells and tissues using the AG RNAex Pro Reagent (AG21102, Accurate Biotechnology, Co., Ltd, Hunan, China) according to the standard protocol. Subsequently, the isolated RNA was reverse-transcribed into cDNA using a reverse-transcriptase kit (Xinbeibio, Shanghai, China). qPCR was conducted with SYBR Green Master mix (Yeason, Shanghai, China) on a ViiATM Real-Time PCR instrument. The relative expression of the target genes was quantified by normalization to GAPDH. Primer sequences were as follows: Gapdh Forward primer: TCTCCTGCGACTTCAACAReverse primer: TGT​AGC​CGT​ATT​CAT​TGT​CA Il1β Forward primer: CCACAGACCTTCCAGGAGAATGReverse primer: GTG​CAG​TTC​AGT​GAT​CGT​ACA​GG Il6 Forward primer: ACAACCTGAACCTTCCAAAGATGReverse primer: TAT​ACC​TCA​AAC​TCC​AAA​AGA​CCA​G Tnfα Forward primer: CACTTCGAAACCTGGGATTCAGReverse primer: GGT​CTC​CAG​ATT​CCA​GAT​GTC​AG Col1a1 Forward primer: TCA​GAG​GCG​AAG​GCA​ACA​GT Reverse primer: CCCCAAGTTCCGGTGTGA Atca2Forward primer: GCT​GAA​GTA​TCC​GAT​AGA​ACA​CG Reverse primer: GGT​CTC​AAA​CAT​AAT​CTG​GGT​CA Il1raForward primer: TCA​GAT​CTG​CAC​TCA​ATG​CC Reverse primer: CTG​GTG​TTT​GAC​CTG​GGA​GT Ifn4 Forward primer: AGGATTTTGGATTCCCCTTGReverse primer: TAT​GTC​CTC​ACA​GCC​AGC​AG Ifnb Forward primer: AGCTCCAAGAAAGGACGAACATReverse primer: GCC​CTG​TAG​GTG​AGG​GTT​GAT​CT Isg15Forward primer: CAG​GAC​GGT​CTT​ACC​CTT​TCC Reverse primer: AGG​CTC​GCT​GCA​GTT​CTG​TAC Cyp7a1 Forward primer: TGGAATAAGGAGAAGGAAAGTAReverse primer: TGT​GTC​CAA​ATG​CCT​TCG​CAG​A Cyp8b1 Forward primer: CCT​CTG​GAC​AAG​GGT​TTT​GTG Reverse primer: GCA​CCG​TGA​AGA​CAT​CCC​C Cyp7b1Forward primer: GGA​GCC​ACG​ACC​CTA​GAT​G Reverse primer: GCC​ATG​CCA​AGA​TAA​GGA​AGC Fxr Forward primer: TGTGAGGGCTGCAAAGGTTTReverse primer: ACA​TCC​CCA​TCT​CTC​TGC​AC Fgf15 Forward primer: GAGGACCAAAACGAACGAAATTReverse primer: ACG​TCC​TTG​ATG​GCA​ATC​G.

### Hematoxylin-eosin (H&E) and sirius red staining

Liver tissues were immediately fixed in 4% paraformaldehyde once resected and then embedded in paraffin after 24–48 h. The embedded tissues were sliced into 4 µm thick sections for histological experiments. The inflammatory response and ballooning were assessed by H&E staining. In addition, collagen accumulation in the liver was evaluated using Sirius red staining. All images were captured using a light microscope, and the positive areas were quantified using ImageJ software (NIH, Bethesda, MD, United States).

### Immunocytochemistry

After deparaffinization, formalin-fixed paraffin-embedded tissues were processed for antigen retrieval using an EDTA antigen retrieval solution (Sangon, Shanghai, China). Immunostaining blocking buffer (Sangon, Shanghai, China) was used to block non-specific antibody binding. The sections were incubated with primary antibodies against α-SMA (1:500, Abcam, 32,575) or F4/80 (1:500, Cell Signaling Technology, 30325S) overnight at 4°C. The slices were then washed and incubated with a horseradish peroxidase-labeled secondary antibody (1:200, Beyotime Biotechnology, Shanghai, China) for 1 h at room temperature. Positive areas were visualized using 3,3-diaminobenzidine tetrahydrochloride and counterstained with hematoxylin. Images were acquired using a fluorescence microscope (Leica). The positive fields were quantified using ImageJ (National Institutes of Health, Bethesda, MD, United States).

### Detection of IFN-β

The levels of interferon-β (IFN-β) in the liver tissues were detected using enzyme-linked immunosorbent assay (ELISA) assay kits (MULTISCIENCES, Hangzhou, China). All procedures followed the manufacturer’s instructions.

### RNA sequencing (RNA-seq)

The expression profiles of liver tissues were detected using RNA-seq. Bulk liver RNA was isolated using the AG RNAex Pro Reagent (AG21102, Accurate Biotechnology, Co., Ltd., Hunan, China) according to the manufacturer’s protocol. RNA was sequenced by Majorbio Bio-Pharm Technology Co., Ltd. (Shanghai, China). The data were analyzed using the online Majorbio Cloud Platform. DESeq was used to analyze differential gene expression. Differentially expressed genes (DEGs) were clustered using hierarchical cluster analysis. Gene ontology (GO) enrichment analysis of DEGs was performed using cluster Profiler.

### Bacterial 16S rRNA sequencing

Stool samples were collected after the mice were sacrificed. The total DNA was extracted using A QIAamp PowerFecal DNA Kit (QIAGEN, Germany). The 16S rRNA V3-V4 region amplified products were subjected to deep sequencing using the Illumina^®^ MiSeq™ II platform. Mothur software was used to remove ambiguous, chimeric, contaminated, and short sequences (length < 350 bp). Operational taxonomic units (OTUs) of high-quality sequences were identified by mapping to sequences in the SILVA database with 97% similarity. Statistical differences in OTU abundance were determined at a threshold of linear discriminant analysis score >3. Alpha and beta diversity analyses were performed using Mothur and Bray–Curtis method, respectively.

### Targeted metabolomics assay

Liver samples were subjected to protein precipitation using acidified and ice-cold MeOH. The supernatant was acquired after vortexing for 60 s, grinding at 55 Hz for 1 min, ultrasonication at 25°C for 10 min, storage at −20°C for 30 min, and centrifugation at 12,000 rpm at 4°C for 10 min. Then, 300 μl of the supernatant was reconstituted in 600 μl ddH_2_O, followed by filtration through a 0.22 μm membrane. Finally, 25% of the 200 μl mixture in 30% MeOH solvent was injected into the LC-MS system for detection. The chromatographic and mass spectrometry conditions for the ultra-performance liquid chromatography system analysis were used as previously described (Yang et al., 2017; Hu et al., 2020).

### Statistical analysis

Statistical analysis was performed using the SPSS software (version 18.0; SPSS Inc., Chicago, IL, United States). Student’s *t*-test was applied for two groups, whereas a one-way analysis of variance was applied for more than two groups. Statistical significance was set at *p* < 0.05.

## Results

### Kuhuang treatment ameliorates hepatic inflammation and fibrosis in BDL mice

**FIGURE 1:**
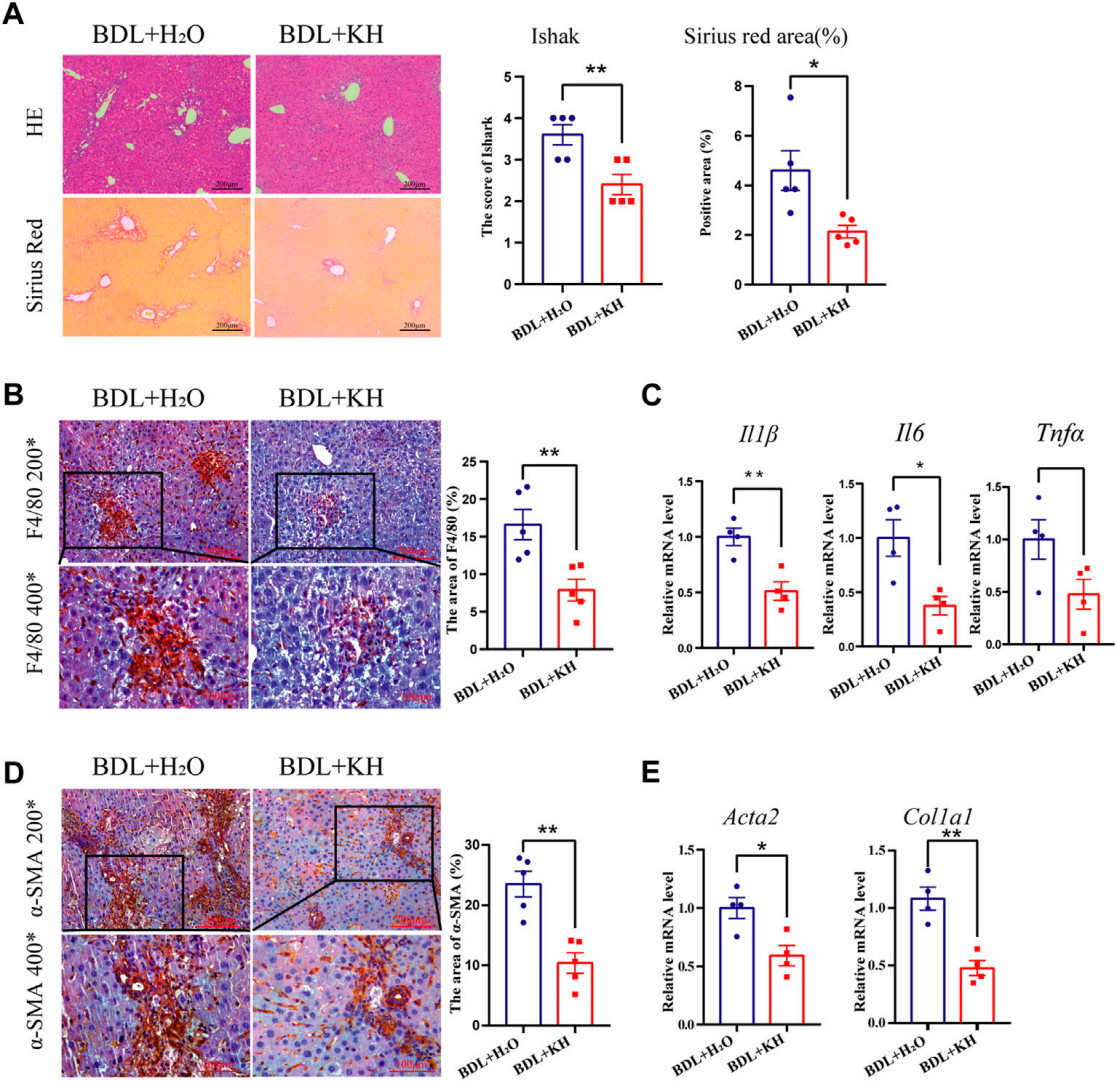
Kuhuang treatment ameliorates hepatic inflammation and fibrosis in BDL mice. **(A)** Representative images of H&E and sirius red staining of liver sections (left panel) of BDL mice. Quantification of Ishak score and Sirius red-positive areas are shown in the right panel. **(B)** Representative images (left panel) and quantification (right panel) of F4/80-positive areas. **(C)** QRT-PCR analyses inflammation-related genes (*Il6*, *Il1β,* and *Tnfα*) in liver tissue. **(D)** Representative images (left panel) and quantification (right panel) of α-SMA positive fibrosis areas. **(E)** QRT-PCR analysis of fibrosis-related genes (*Col1a1* and *Acta2*) in liver tissue. The data are presented as mean ± SEM. N = 4–5 per group. The data were analyzed with Student’s *t*-test. *p < 0.05, **p < 0.01, ***p < 0.001. Abbreviations, KH, Kuhuang; BDL, bile duct ligation.

Mice that underwent BDL surgery to induce cholestatic liver fibrosis were divided into two groups: Kuhuang and H_2_O (control group). H&E staining showed less inflammatory cell infiltration and centrilobular necrosis in the liver of the Kuhuang group than in control (Figure 1A). Fewer macrophages were observed, and hepatic mRNA expression of *Il6, Il1β,* and *Tnfα* was downregulated in the liver of Kuhuang mice, indicating that Kuhuang treatment attenuated the liver inflammatory response (Figures 1B, C). In addition, Sirius red staining and IHC for α-SMA (Figures 1A, D) revealed that the hepatic profibrotic area was significantly reduced in the Kuhuang group. The mRNA levels of profibrogenic genes (*Acta2* and *Col1a1*) were downregulated upon Kuhuang treatment (Figure 1E). These results indicate that Kuhuang treatment ameliorated hepatic inflammation and liver fibrosis in BDL mice.

### Kuhuang treatment ameliorates hepatic inflammation and fibrosis in DDC mice

**FIGURE 2:**
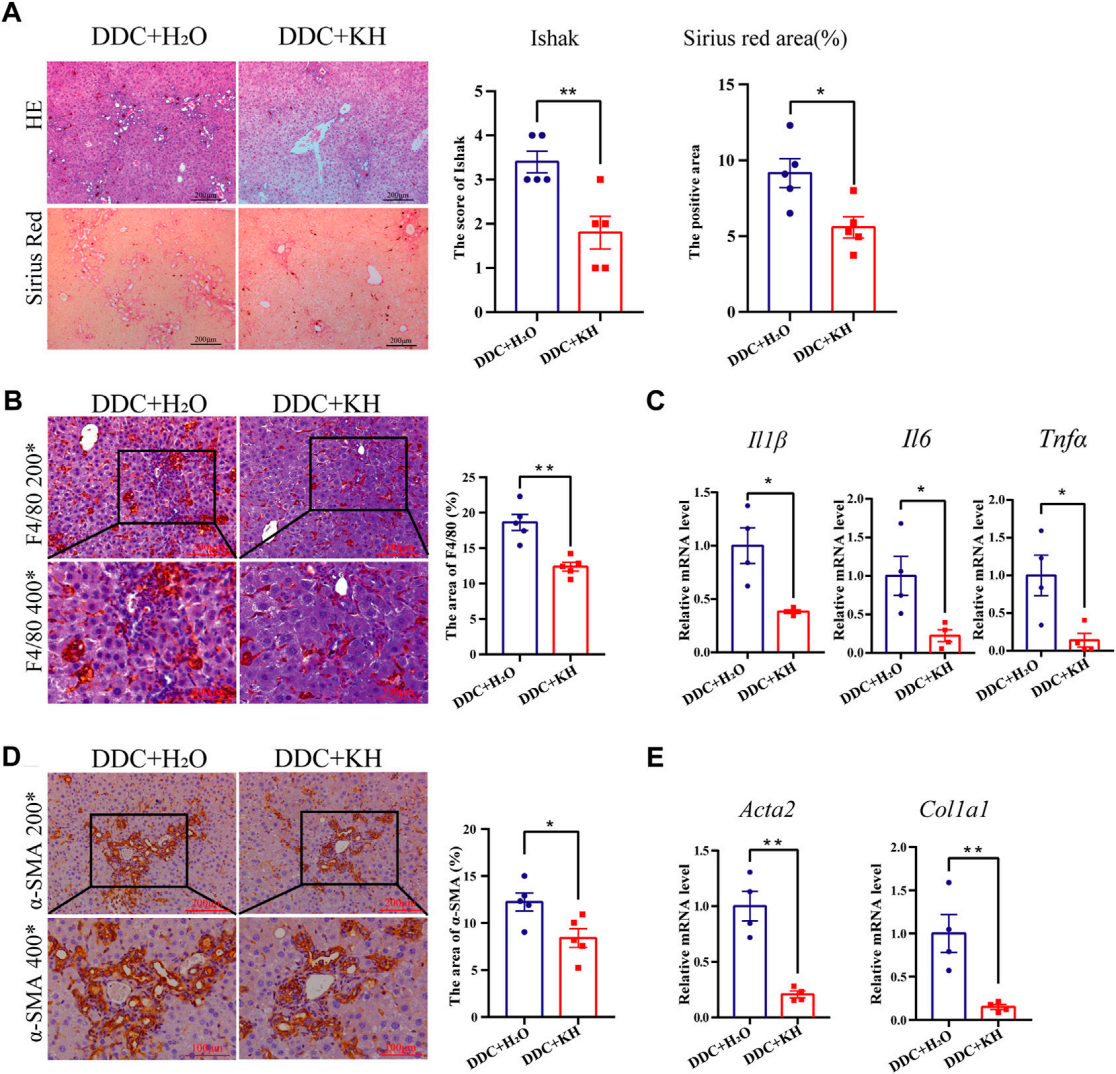
Kuhuang treatment ameliorates hepatic inflammation and fibrosis in DDC mice. **(A)** Representative images of H&E and Sirius red staining of liver sections (left panel) of DDC mice. Quantification of Ishak score and Sirius red-positive areas are shown in the right panel. **(B)** Representative images (left panel) and quantification (right panel) of F4/80-positive areas. **(C)** QRT-PCR analysis of inflammation-related genes (*Il6*, *Il1β,* and *Tnfα*) in liver tissue. **(D)** Representative images (left panel) and quantification (right panel) of α-SMA positive fibrosis areas. **(E)** QRT-PCR analysis of fibrosis-related genes (*Col1a1* and *Acta2*) in liver tissue. The data are presented as mean ± SEM. N = 4–5 per group. The data were analyzed with Student’s *t*-test. *p < 0.05, **p < 0.01, ***p < 0.001. Abbreviations: KH, Kuhuang; DDC, 3,5-diethoxycarbonyl-1,4-dihydro-collidi​ne.

Cholestatic liver injury was also induced in mice by a 4-week DDC diet and oral administration of either Kuhuang or H_2_O. H&E staining showed less inflammatory cell infiltration and centrilobular necrosis in the liver of the Kuhuang group than in control (Figure 2A). Fewer macrophages were observed, and hepatic mRNA expression of *Il6, Il1β,* and *Tnfα* was downregulated in the liver of Kuhuang mice, indicating that Kuhuang treatment attenuated the liver inflammatory response (Figures 2B, C). In addition, Sirius red staining and IHC for α-SMA revealed that the hepatic profibrotic area was significantly reduced in the Kuhuang group (Figures 2A, D). The mRNA levels of profibrogenic genes (*Acta2* and *Col1a1*) were downregulated upon Kuhuang treatment (Figure 2E). These results indicate that Kuhuang treatment ameliorates hepatic inflammation and liver fibrosis in DDC mice.

### Kuhuang treatment upregulates interferon-related pathway

**FIGURE 3:**
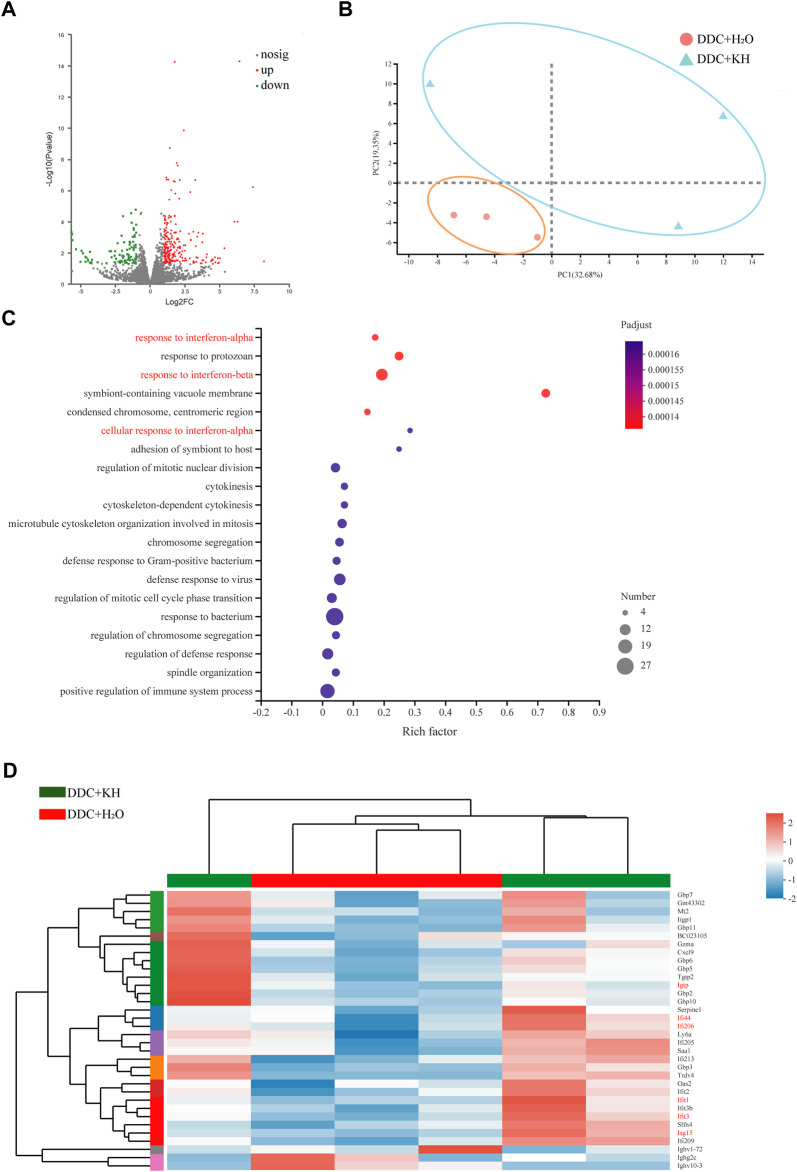
Kuhuang treatment upregulates interferon-related pathways in DDC mice. **(A)** Volcano plot of differentially expressed genes (612 upregulated and 370 downregulated) between DDC + KH and DDC + H_2_O group. **(B)** PCA of gene expression profiles of DDC + KH and DDC + H_2_O group. **(C)** GO enrichment of genes differentially expressed between DDC + KH and DDC + H_2_O group. **(D)** Heatmap of genes differentially expressed between DDC + KH and DDC + H_2_O group. Abbreviations: KH, Kuhuang; DDC, 3,5-diethoxycarbonyl-1,4-dihydro-collidine.

We performed RNA-seq analysis of the liver tissues from Kuhuang and control groups in DDC mice to explore the potential mechanisms involved in alleviating hepatic inflammation and liver fibrosis by Kuhuang treatment. A total of 982 genes were differentially expressed, of which 612 genes were upregulated and 370 were downregulated in Kuhuang-treated mice compared to the control (Figure 3A). Principal component analysis (PCA) identified a distinct gene expression pattern between the two groups, with PC1 explaining 32.68% of the variation and PC2 explaining 19.35% of the variation (Figure 3B). Gene Ontology (GO) enrichment analysis revealed that DEGs were enriched in pathways associated with interferon regulation, such as response to interferon-α, response to interferon-β, and cellular response to interferon-α (Figure 3C). As shown in Figure 3D, genes involved in interferon-related pathways, such as *Igtp*, *Iif44*, *Iif206*, *Ifit1*, *Ifit3,* and *Isg15*, were significantly upregulated after Kuhuang treatment (Figure 3D). These data indicate that Kuhuang treatment altered the hepatic gene expression pattern, especially related to the interferon pathway.

### Kuhuang treatment alters gut microbiota composition and hepatic BAs

**FIGURE 4:**
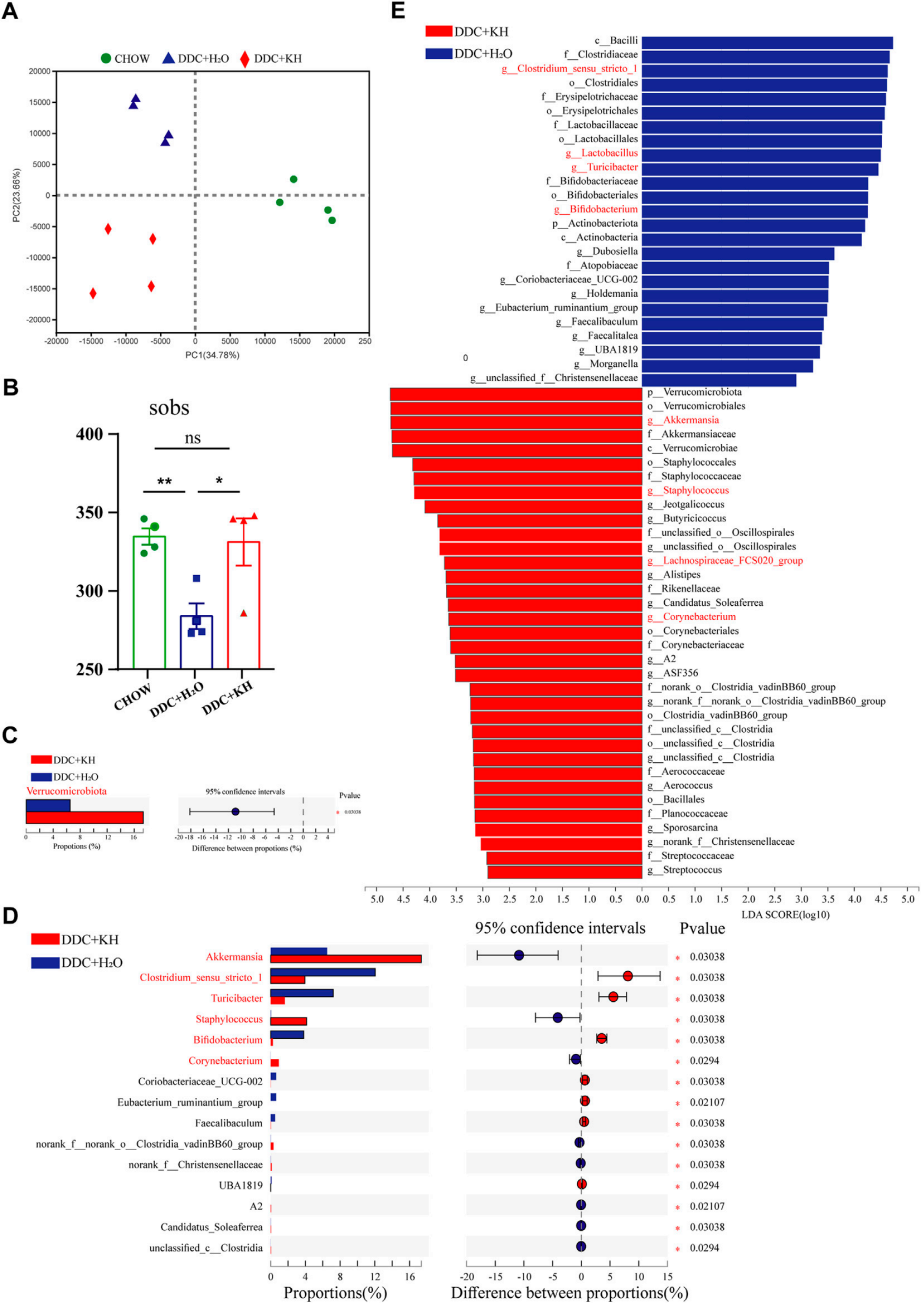
Kuhuang treatment altered gut microbiota composition in DDC mice. **(A)** Bray–Curtis distance-based PCOA of gut microbiota diversity of Chow, DDC + KH, and DDC + H_2_O groups. **(B)** Sobs in Chow, DDC + KH, and DDC + H_2_O groups. The data are presented as mean ± SEM. N = 4–5 per group. The data were analyzed with Student’s *t*-test. *p < 0.05, **p < 0.01, ***p < 0.001. **(C)** Gut microbiota with statistically differential abundance at phylum level between DDC + KH and DDC + H_2_O groups. **(D)** Gut microbiota with statistically differential abundance at genus level between DDC + KH and DDC + H_2_O groups. **(E)** LEfSe analysis of differentially abundant microbes between DDC + KH and DDC + H_2_O groups. Abbreviations: KH, Kuhuang; DDC, 3,5-diethoxycarbonyl-1,4-dihydro-collidi​ne.

Next, we explored the influence of Kuhuang treatment on the composition of gut microbiota through 16S rRNA sequencing of fecal samples from DDC mice treated with Kuhuang or H_2_O, as well as from mice fed a chow diet. Principal coordinate analysis (PCOA) showed a distinct deviation along PC1 (explaining 34.78% of variation) and PC2 (explaining 23.66% of variation) among groups, suggesting that either the DDC diet or Kuhuang treatment would lead to alterations in gut microbiota (Figure 4A). Notably, Kuhuang treatment increased the diversity of gut microbiota (as indicated by sobs), which was significantly decreased in DDC mice compared with mice fed a chow diet, implying that Kuhuang might be able to rescue gut microbiota dysbiosis induced by the DDC diet (Figure 4B). Further analysis showed that, compared with DDC mice treated with H_2_O, the abundance of *Verrucomicrobiota* was remarkably higher at the phylum level in mice treated with Kuhuang (Figure 4C). At the genus level, the abundance of *Akkermansia*, *Staphylococcus,* and *Corynebacterium* was significantly higher, whereas that of *Clostridium_sensu_stricto_1*, *Turicibacter,* and *Bifidobacterium* was significantly lower in mice treated with Kuhuang (Figure 4D). LEfSe analysis revealed differential gut microbiota between mice treated with Kuhuang and with H_2_O. As shown in Figure 4E, *Akkermansia*, *Staphylococcus*, *Corynebacterium*, and Lachnospiraceae *FCS020 group* were more abundant, while *Clostridium*_*-*_ *sensu*_-_ *stricto*_*-*_ *1*, *Turicibacter*, *Bifidobacterium,* and *Lactobacillus* were less abundant upon Kuhuang treatment. These results indicate that Kuhuang treatment altered the composition of the gut microbiota and rescued dysbiosis induced by the DDC diet.

**FIGURE 5:**
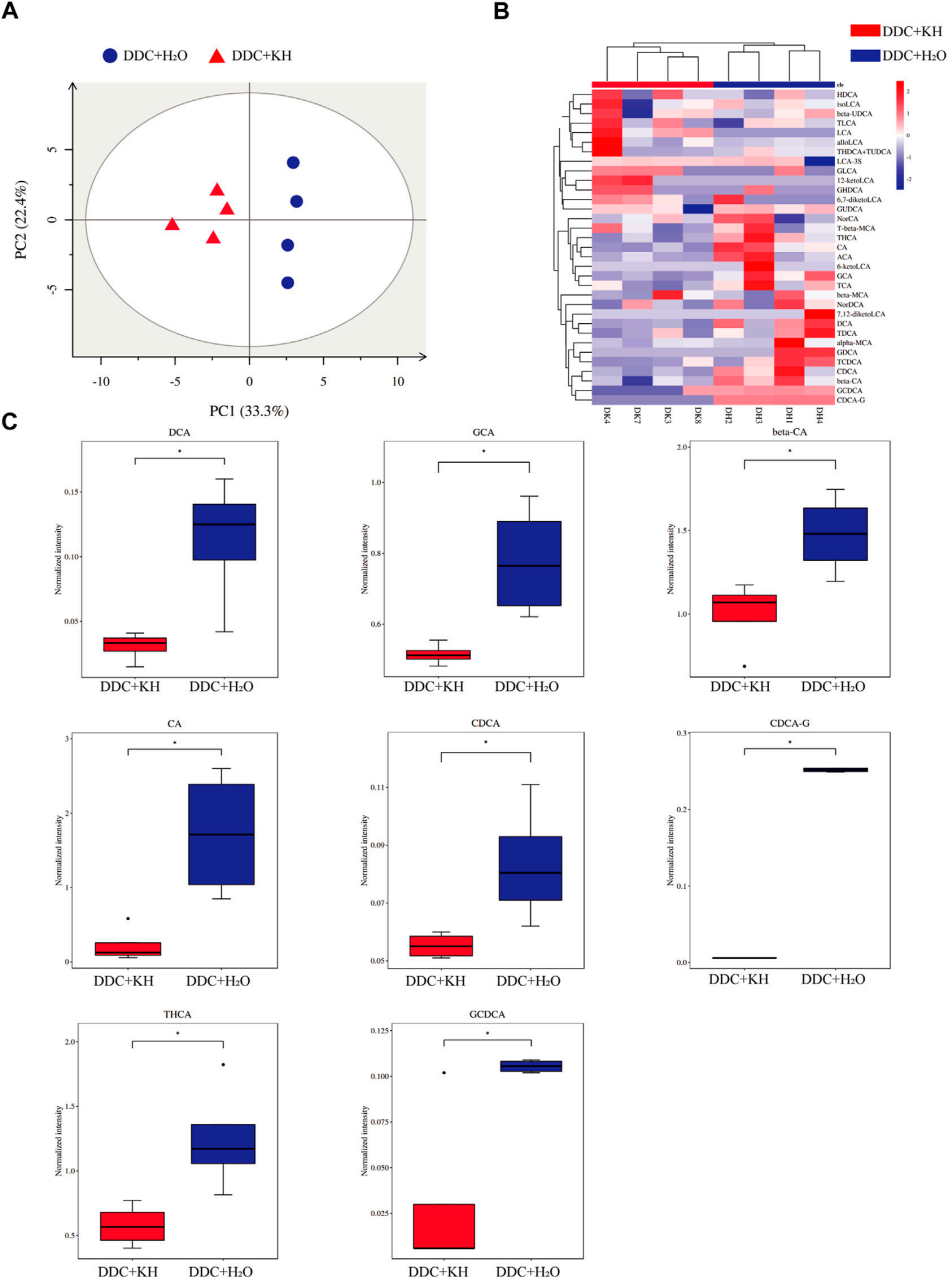
Kuhuang treatment altered hepatic BA levels in DDC mice. **(A)** PCA of hepatic BA composition of DDC + KH and DDC + H_2_O groups. **(B)** Heatmap of hepatic BAs with statistically different abundance between DDC + KH and DDC + H_2_O groups. **(C)** Histogram of normalized intensity of DCA, GCA, beta-CA, CA, CDCA, CDCA-G, THCA, and GCDCA in DDC + KH and DDC + H_2_O groups. The data are presented as mean ± SEM. N = 4–5 per group. The data were analyzed with Student’s *t*-test. *p < 0.05, **p < 0.01, ***p < 0.001. Abbreviations: KH, Kuhuang; DDC, 3,5-diethoxycarbonyl-1,4-dihydro-collidi​ne.

Furthermore, the composition of hepatic bile acids was examined using a targeted metabolomic assay in mice fed a DDC diet. PCA analysis showed distinct clustering along PC1 (explaining 33.3% of the variation) and PC2 (explaining 22.4% of the variation) between mice treated with Kuhuang and H_2_O (Figure 5A). Remarkably, Kuhuang treatment significantly decreased the levels of both primary bile acids (CA, CDCA, and GCDCA) and secondary bile acids (DCA, GCA, CDCA-G, and THCA), suggesting that Kuhuang treatment may reduce BAs in the liver (Figures 5B, C).

### Correlation of gut microbiota abundance and the expression of hepatic genes

**FIGURE 6:**
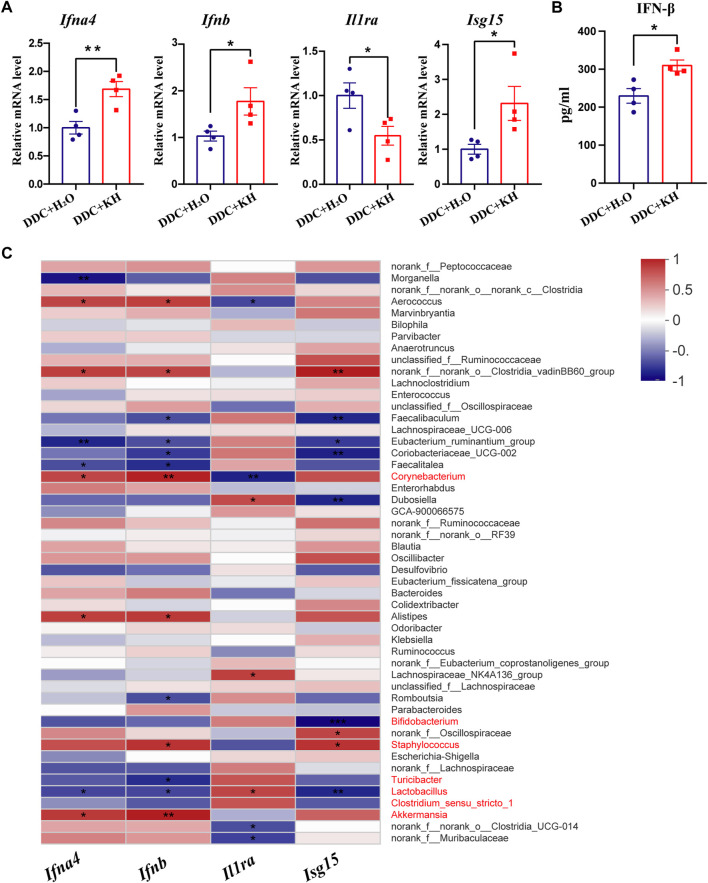
Correlation of gut microbiota abundance and hepatic IFN-related genes in DDC mice. **(A)** QRT-PCR analysis of IFN-related genes (*Ifna4*, *Ifnb, Il1ra,* and *Isg15)* in liver tissue. **(B)** ELISA analysis of hepatic IFN-β level. The data are presented as mean ± SEM. N = 4–5 per group. The data were analyzed with Student’s *t*-test. *p < 0.05, **p < 0.01, ***p < 0.001. **(C)** Correlation analysis between the expression levels of IFN-related genes and gut microbiota abundance. Abbreviations: KH, Kuhuang; DDC, 3,5-diethoxycarbonyl-1,4-dihydro-collidi​ne.

Based on our findings from RNA-seq analysis, we further validated the hepatic mRNA expression levels of interferon-related genes (*Ifna4*, *Ifnb*, *Isg15,* and *Il1ra*) by quantitative RT-PCR. Kuhuang treatment significantly increased the hepatic mRNA expression levels of *Ifna4*, *Ifnb,* and *Isg15* and decreased that of *Il1ra* (Figure 6A). In addition, significant upregulation of IFNβ levels in the liver tissue of mice treated with Kuhuang was confirmed by ELISA (Figure 6B). Correlation analysis showed that the abundance of gut *Akkermansia, Corynebacterium,* and *Staphylococcus* was positively correlated with the hepatic mRNA expression levels of *Ifna4*, *Ifnb,* and *Isg15,* and that of *Corynebacterium* was negatively correlated with *Il1ra* expression level. The abundance of *Lactobacillus*, *Clostridium-sensu-stricto-1, Bifidobacterium,* and *Turicibacter* was negatively correlated with *Ifna4*, *Ifnb,* and *Isg15* expression levels, and that of *Lactobacillus* was positively correlated with *Il1ra* expression level (Figure 6C). These results implied that the gut microbiota might participate in the regulation of interferon-related genes, which is consistent with previous investigations demonstrating that microbiota-derived stimulators could prompt type I interferon production by targeting interferon-related genes (Lam et al., 2021).

**FIGURE 7:**
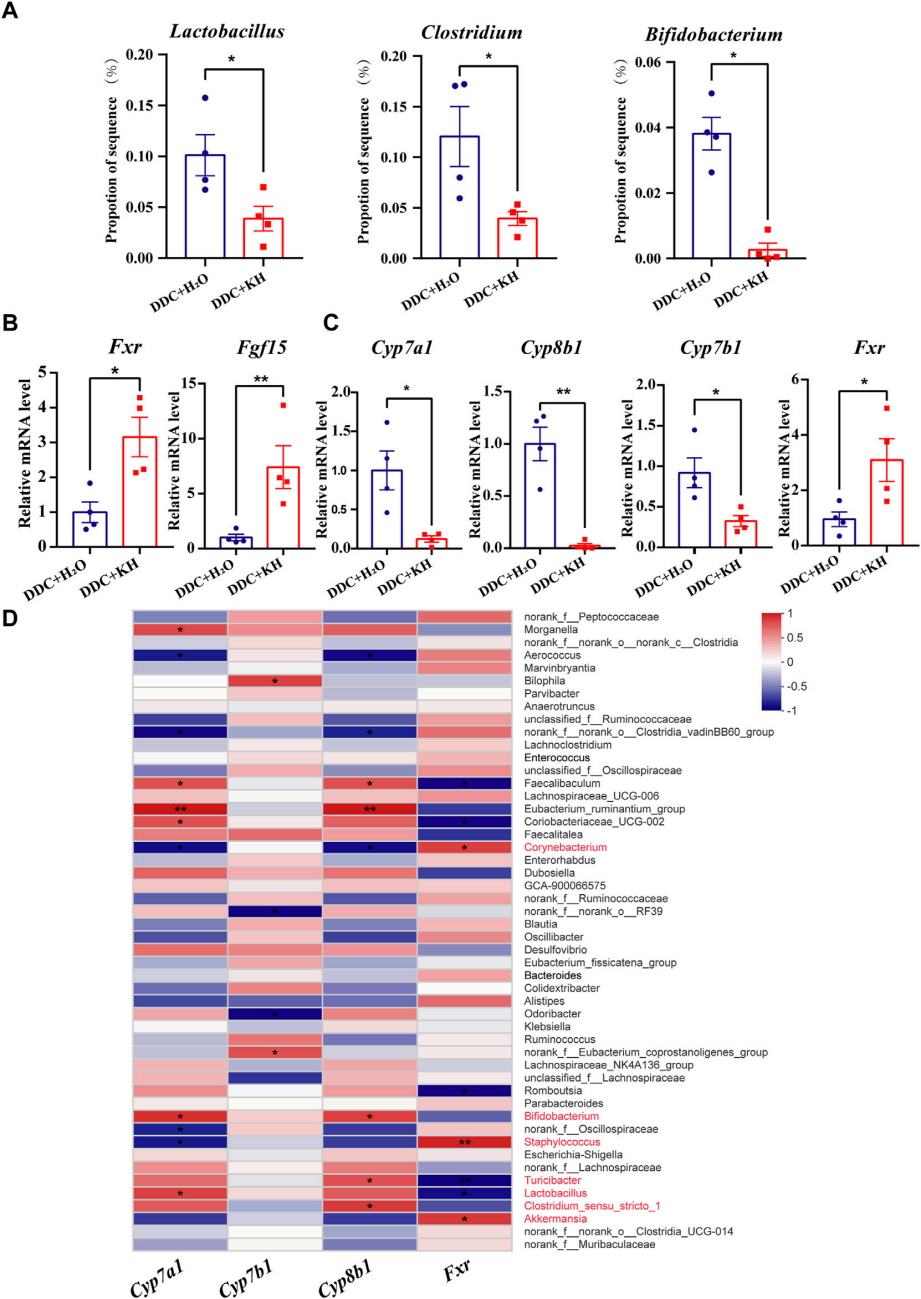
Correlation of gut microbiota abundance and hepatic BA synthesis-related genes in DDC mice. **(A)** Histogram of *Lactobacillus, Clostridium,* and *Bifidobacterium* proportions in gut microbiota in DDC + KH and DDC + H_2_O groups. **(B)** QRT-PCR analysis of *Fxr* and *Fgf15* in ileum tissue. **(C)** QRT-PCR analysis of BA synthesis-related genes (*Cyp7a1, Cyp7b1, Cyp8b1*and *Fxr*) in liver tissue. The data are presented as mean ± SEM. N = 4–5 per group. The data were analyzed with Student’s *t*-test. *p < 0.05, **p < 0.01, ***p < 0.001. **(D)** Correlation analysis between the expression levels of BA synthesis-related genes and gut microbiota abundance. Abbreviations: KH, Kuhuang; DDC, 3,5-diethoxycarbonyl-1,4-dihydro-collidi​ne.

By analyzing the results of 16S rRNA sequencing, we found that the abundance of bile salt hydrolase (BSH)-producing gut microbiota, such as *Lactobacillus*, *Clostridium,* and *Bifidobacterium,* were less abundant in DDC mice treated with Kuhuang than in those treated with H_2_O (Figure 7A). We also found that compared with DDC mice treated with H_2_O, the mRNA expression of *Fxr* and *Fgf15* was significantly upregulated in the ileum of DDC mice treated with Kuhuang (Figure 7B). Furthermore, hepatic mRNA expression of BA synthesis-related genes (*Cyp7a1*, *Cyp8b1,* and *Cyp7b1*) was downregulated, whereas that of *Fxr* was upregulated significantly in DDC mice treated with Kuhuang (Figure 7C). Correlation analysis showed that the abundance of gut *Bifidobacterium*, *Clostridium*_*-*_ *sensu*_*-*_ *stricto*_*-*_ *1*, *Turicibacter,* and *Lactobacillus* was positively correlated with BA synthesis-related genes (*Cyp7a1*, *Cyp8b1,* and *Cyp7b1*), and *Turicibacter* and *Lactobacillus* were negatively correlated with *Fxr*. In addition, the abundances of gut *Corynebacterium*, *Staphylococcus*, and *Akkermansia* were negatively correlated with BA synthesis-related genes (*Cyp7a1*, *Cyp8b1,* and *Cyp7b1*) and positively correlated with *Fxr* (Figure 7D). These data suggest that Kuhuang-induced gut microbiota alteration may also regulate hepatic genes related to BA synthesis.

## Discussion

Kuhuang has been reported to relieve jaundice symptoms and reduce elevated liver enzyme levels in clinical practice (Zhao et al., 2017). In this study, we found that Kuhuang treatment significantly alleviates the progression of liver fibrosis in both the DDC and BDL mouse models. In addition, Kuhuang treatment mitigated liver inflammation. These results suggest that Kuhuang is a potential drug for treating liver fibrosis. The abundance of gut *Firmicutes* and *Bacteroidetes* was decreased, and that of *Proteobacteria* and *Fusobacteria* is increased in patients with liver cirrhosis, suggesting that the gut microbiota played an important role in the development of liver fibrosis (Preveden et al., 2017). Our study showed that Kuhuang treatment significantly altered gut microbiota composition with an increased abundance of *Akkermansia, Staphylococcus, Corynebacterium*, and Lachnospiraceae *FCS020 group* and decreased abundance of *Turicibacter*, *Clostridium*_*-*_ *sensu*_-_ *stricto*_*-*_ *1*, *Bifidobacterium,* and *Lactobacillus* in a mouse model of liver fibrosis. *Clostridium_sensu_stricto_1* was known to be enriched with increased severity of liver fibrosis (Xiang et al., 2022). Furthermore, decreased abundance of gut *Clostridium_sensu_stricto-1* ameliorated liver inflammation in mice with non-alcoholic steatohepatitis (Rom et al., 2020) or alcoholic liver disease (Guo et al., 2022a). Thus, we hypothesize that Kuhuang treatment attenuates liver fibrosis through *Clostridium*-mediated alleviation of inflammation. The Kuhuang treatment also increased the abundance of Lachnospiraceae *FCS020 group*. Lachnospiraceae participated in the synthesis of short-chain fatty acids (SCFAs) (Louis and Flint, 2017), which was critical in ensuring the integrity of the intestinal mucosal barrier (Parada Venegas et al., 2019). Our previous studies showed that disruption of the intestinal barrier was associated with the severity of liver fibrosis (Shen et al., 2021). Therefore, Kuhuang treatment may improve liver fibrosis through gut *Lachnospiraceae-*induced SCFAs to enhance the integrity of the intestinal mucosal barrier. In addition, *Akkermansia* played a similar role in protecting the intestinal barrier, reducing plasma endotoxin levels, and inhibiting intestinal inflammation and metabolic disorders (Depommier et al., 2019). Thus, Kuhuang may alleviate liver inflammation and fibrosis by increasing the abundance of *Akkermansia*, which inhibits intestinal inflammation and decreases endotoxin levels. In short, altered gut composition upon Kuhuang treatment may improve liver fibrosis by preventing intestinal endotoxins from entering the liver.

In addition to the gut microbiota, Kuhuang treatment altered the gene expression pattern in the liver. The interferon signaling-related genes (*Ifna4*, *Ifnb,* and *Isg15*) were significantly upregulated. Type I interferon protected against liver injury and inhibits inflammatory responses (Petrasek et al., 2011). In addition, α-interferon could effectively alleviate the progression of liver fibrosis in patients with chronic hepatitis C (Serejo et al., 2001). Furthermore, correlation analysis indicated that the abundance of *Akkermansia, Corynebacterium,* and *Staphylococcus* increased upon Kuhuang treatment and was positively correlated with the hepatic expression of *Ifna4*, *Ifnb,* and *Isg15*. Interestingly, the abundance of *Lactobacillus*, *Clostridium-sensu-stricto-1, Bifidobacterium,* and *Turicibacter* decreased upon Kuhuang treatment and was negatively correlated with *Ifna4*, *Ifnb,* and *Isg15* expression. The results suggest that Kuhuang treatment may inhibit liver inflammation and fibrosis by regulating the liver’s interferon signaling (activating type I interferon), mediated by modulating microbiota composition. Lam et al. (2021) showed that *Akkermansia* produced c-di-AMP and induced the production of type I interferon. Type I interferon might alleviate liver fibrosis by inhibiting HSC activation and reducing immunological reactions (Tanabe et al., 2007; Shimozono et al., 2015). Our study found that Kuhuang treatment reduced the hepatic expression levels of profibrotic genes (*Acta2* and *Col1a1*) and inflammatory cytokines (*Il6*, *Il1β*, and *Tnfα*) while upregulating interferon signaling-related genes (*Ifna4*, *Ifnb,* and *Isg15*). Thus, we hypothesize that Kuhuang may alleviate liver inflammation and fibrosis by increasing the abundance of *Akkermansia*, which leads to activating the type I interferon pathway in the liver.

Furthermore, our results showed that Kuhuang treatment significantly decreased the conjugated and non-conjugated BAs in the profibrotic liver. It significantly decreased the hepatic mRNA expression levels of *Cyp7a1* and *Cyp8b1*, cytochrome P450 enzymes that are crucial for BA synthesis in the classical pathway. Notably, the classical pathway accounts for approximately 75% of BA synthesis (Thomas et al., 2008). In addition, Kuhuang treatment significantly decreased the mRNA expression level of *Cyp7b1* in the liver, the key enzyme that regulates BA synthesis *via* an alternative pathway (Russell, 2003). Kuhuang treatment also significantly increased hepatic mRNA expression level of *Fxr.* In the liver, Fxr substantially inhibits BA synthesis (Lu et al., 2000). Thus, Kuhuang treatment may suppress BA retention by inhibiting Fxr-mediated BA synthesis in the liver. Cholestasis in the liver is an important factor contributing to the development of liver fibrosis (Ghonem et al., 2015). Cholestasis and destruction of the bile duct damage the bile duct cells and hepatocytes, further promoting the progression of liver fibrosis. Therefore, Kuhuang treatment may attenuate liver inflammation and fibrosis by inhibiting BA synthesis. In addition, the abundance of gut *Turicibacter* and *Lactobacillus*, which decreased upon Kuhuang treatment, was negatively correlated with the hepatic expression of *Fxr*. Meanwhile, the abundance of *Corynebacterium, Staphylococcus*, and *Akkermansia*, which increased upon Kuhuang treatment, was positively correlated with *Fxr* expression in the liver, suggesting that gut microbiota alteration may eventually result in the inhibition of hepatic Fxr-mediated BA synthesis. In addition, we found that Kuhuang treatment significantly reduced the abundance of BSH-producing *Lactobacillus*, *Clostridium,* and *Bifidobacterium*, which might result in increased levels of intestinal conjugated BAs, such as LCA and TCA, activators of Fxr (Wahlström et al., 2016; Xiang et al., 2021; Münzker et al., 2022). We observed that the expression levels of *Fxr* and *Fgf15* in the ileum significantly increased after Kuhuang treatment. Fxr-FGF15 activation in the ileum can further inhibit the hepatic expression of BA synthesis-related genes and BA synthesis in the liver (Stofan and Guo, 2020; Katafuchi and Makishima, 2022). In addition, correlation analysis showed that the abundance of BSH-producing *Lactobacillus, Clostridium,* and *Bifidobacterium* was positively correlated with the expression levels of genes related to BA synthesis (*Cyp7a1, Cyp7b1* or *Cyp8b1*). Thus, we hypothesize that Kuhuang treatment may inhibit hepatic BA synthesis by activating the Fxr-FGF15 in the ileum mediated by altering the abundance of BSH-producing gut microbiota.

**FIGURE 8:**
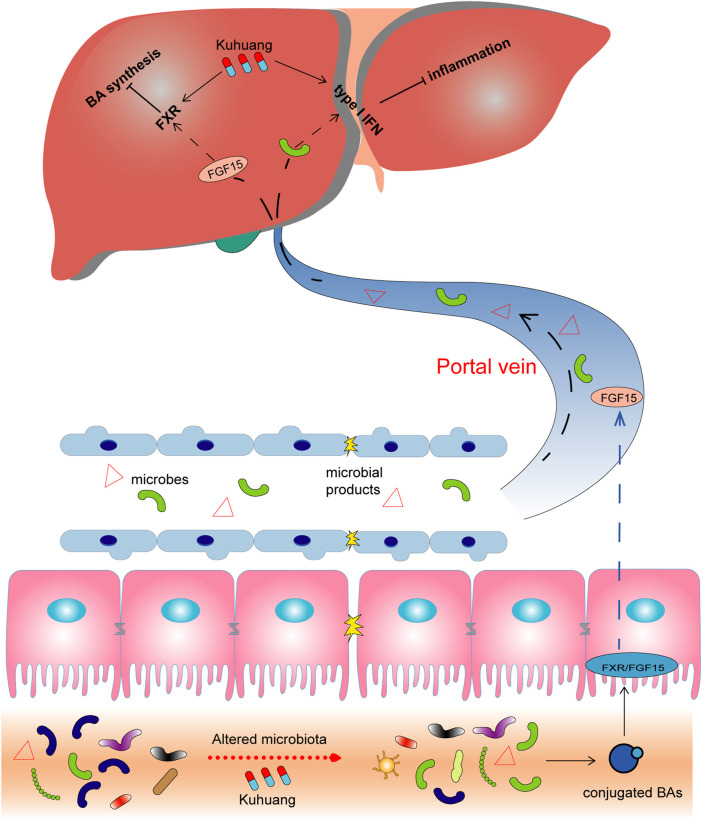
Schematic illustration of potential mechanisms of Kuhuang. Kuhuang may inhibit BA synthesis and inflammation responses in the liver by upregulating interferon signaling-related genes while downregulating genes associated with BA synthesis. Kuhuang also alters the composition of gut microbiota, consequently benefiting the maintenance of the intestinal barrier and increasing the level of conjugated BAs. Conjugated BAs activate intestinal Fxr/FGF15 signaling, followed by hepatic Fxr activation, which further inhibits BA synthesis in the liver. In addition, gut microbes and microbial products that translocate into the liver can also inhibit hepatic inflammation.

In summary, our study indicates that Kuhuang attenuates inflammation and BA accumulation and eventually improves fibrosis in a cholestatic mouse model. Kuhuang treatment alters the expression of interferon signaling- and BA synthesis-related genes and decreases hepatic BA levels. Furthermore, alterations in the gut microbiota induced by Kuhuang, especially the increased abundance of interferon-inducing *Akkermansia* and decreased abundance of BSH-producing *Lactobacillus*, *Clostridium,* and *Bifidobacterium*, play potential roles in alleviating liver inflammation and BA accumulation by inhibiting intestinal endotoxin, activating hepatic interferon signaling, and suppressing hepatic BA synthesis (Figure 8). Nevertheless, more experimental and clinical evidence is needed before Kuhuang can be applied to treat liver fibrosis.

## Data Availability

The datasets presented in this study can be found in online repositories. The names of the repository/repositories and accession number(s) can be found below: https://www.ncbi.nlm.nih.gov/bioproject; PRJNA893596.

## Glossary

### The following abbreviations are used in this document:

Acta2: actin alpha 2 smooth muscle
BA: bile acid
BDL: bile duct ligation
BSH: bile salt hydrolase
CKI: compound kushen injection
Col1a1: collagen type 1 α
CYP7a1: cytochrome P450 family 7 subfamily A member 1
CYP7b1: cytochrome P450 family 7 subfamily B member 1
CYP8b1: cytochrome P450 family 8 subfamily B member 1
DDC: 3,5-diethoxycarbonyl-1,4-dihydro-collidine
DEG: differentially expressed gene
ELISA: enzyme-linked immunosorbent assay
FGF 15: fibroblast growth factor 15
Fxr: farnesol X receptor
GAPDH: glyceraldehyde-3-phosphate dehydrogenase
GO: gene ontology
H&E: hematoxylin and eosin
HSC: hepatic stellate cells
Ifna4: interferon alpha 4
IFN-β: interferon-β
IL1 β: interleukin 1β
Il1ra: interleukin 1 receptor antagonist
IL6: interleukin 6
Isg15: ISG15 ubiquitin-like modifier
OTUs: operational taxonomic units
PCA: principal component analysis
QRT-PCR: quantitative real-time polymerase chain reaction
RNA-seq: RNA sequencing
SCFA: short-chain fatty acid
TNF: α tumor necrosis factor α
